# Habitat and Anthropogenic Determinants of Otter Presence in the Karnali River, Western Nepal

**DOI:** 10.1002/ece3.73865

**Published:** 2026-06-14

**Authors:** Madan Acharya, Asmit Subba, Naresh Pandey, Laxman Khanal

**Affiliations:** ^1^ Central Department of Zoology, Institute of Science and Technology Tribhuvan University Kathmandu Nepal; ^2^ Conservation Himalaya Kathmandu Nepal

**Keywords:** anthropogenic disturbance, Karnali River, otter conservation, riparian ecosystem, threatened taxa

## Abstract

Freshwater ecosystems support global biodiversity but face mounting anthropogenic threats. Otters (Carnivora: Mustelidae: Lutrinae) are semi‐aquatic apex predators that are vital for maintaining ecosystem balance, yet they are undergoing widespread global declines. This study assessed otter habitat use and conservation threats along the Western Bend of the Karnali River in western Nepal. From 102 transects (100 m long, 10 m wide) surveyed (October–December 2024), otters were detected at 35 sites based on direct and indirect evidence. Model‐averaged logistic regression identified six significant predictors of occurrence: negative associations with house density (OR = 0.001, *p* < 0.001), river width (OR = 0.075, *p* = 0.009), and water current velocity (OR = 0.293, *p* = 0.041); and positive associations with moderate riparian vegetation (26%–50%; OR = 158.1, *p* = 0.003), small stone substrate (OR = 3.85, *p* = 0.002), and absence of dogs (OR = 20.94, *p* = 0.005). The composite Human Disturbance Index showed a non‐significant negative effect, indicating limited tolerance to general human activity but strong avoidance of settlements and dogs. Our findings confirm otter persistence in this river stretch outside the protected area network of the country and emphasize targeted conservation, particularly dog management, reduced settlement pressure, and riparian habitat maintenance, to secure these keystone species in human‐modified freshwater systems.

## Graphical abstract

Despite increasing human pressures, otters persist along the Western Bend of the Karnali River. Occurrence of otters decreases with higher house density and presence of dogs. The study indicates otters tolerate some general human activity but strongly avoid settlements and dogs, highlighting the need for dog management, reduced settlement pressure, and riparian habitat maintenance for their conservation.

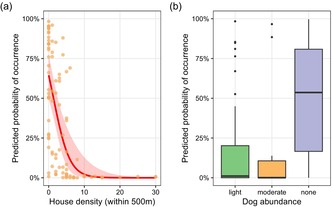

## 1 Introduction

Freshwater ecosystems, which harbor nearly one‐third of the world's vertebrate species, are critical to global biodiversity. However, human activities have increasingly degraded these habitats, severely impacting aquatic life (Dudgeon et al. 2006). As semi‐aquatic apex predators, otters (Carnivora: Mustelidae: Lutrinae) play a key role in maintaining the ecological integrity of freshwater systems by supporting both biodiversity and ecosystem health (Kathariya et al. 2023; Kruuk 2006). Freshwater otters are increasingly threatened by habitat fragmentation, chemical contamination, and direct human interactions, including illegal poaching and incidental capture in fishing gear, which collectively degrade habitat quality and pose significant risks to population viability (Khoo et al. 2021; Loy et al. 2022).

Of the fourteen otter species found globally, three‐ the Eurasian otter ( *Lutra lutra* ), smooth‐coated otter ( *Lutrogale perspicillata* ), and Asian small‐clawed otter ( *Aonyx cinereus* ), occur in Nepal (de Ferran et al. 2024; Jha et al. 2020). Remarkably, the Asian small‐clawed otter, which had not been recorded in the country since 1839 (Acharya et al. 2023), was recently rediscovered in far western Nepal after 185 years (Shrestha et al. 2025). The Karnali River Basin in western Nepal, characterized by its extensive network of rivers, tributaries, and wetlands, provides critical habitat for all three species (Acharya and Rajbhandari 2011; Jha et al. 2020; Kathariya et al. 2023). Globally, the Eurasian otter is listed as Near Threatened, while the smooth‐coated and Asian small‐clawed otters are categorized as Vulnerable on the IUCN Red List of Threatened Species (Khoo et al. 2021; Loy et al. 2022; Wright et al. 2021). Otters inhabit a diverse range of habitats such as rivers, lakes, marshes, waterways and the coastline (Kruuk 2006; Mason and Macdonald 1986). They have been recorded at elevations above 4000 m in Tibet (Mason and Macdonald 1986), while within the Karnali River they are recorded from the upstream reaches in Mugu (approximately 1200–1600 m above sea level) down to the lowland plains of Bardia (approximately 152–200 m above sea level) (Acharya et al. 2023; Gwachha et al. 2023; Jha 2018; Kathariya et al. 2023; Shrestha et al. 2023).

Their populations face major threats from habitat destruction, water pollution, human encroachment, and unsustainable fishing (Kafle 2011; Kathariya et al. 2023). Human activities such as pollution, habitat degradation, and water management projects like dam construction also pose serious risks to aquatic organisms (Shrestha et al. 2022). As an example, agricultural runoff and industrial effluent can pollute water and lead to a decrease in water quality and prey reserves (Awasthi et al. 2024; Cook et al. 2022), while sand mining and deforestation contribute to habitat fragmentation (Kathariya et al. 2023; Shrestha et al. 2022). Studies across Asia indicate that otters generally prefer secluded areas with minimal human disturbance (Basnet et al. 2020; Gupta et al. 2020). Nevertheless, they have shown resilience in modified landscapes (Lee 1996; Theng and Sivasothi 2016), adaptability in habitat choice, and a capacity to recover from population declines (Weinberger et al. 2016). The three species exhibit variable habitat preferences, but their niches and distribution ranges show some overlap (Hussain et al. 2011; Kruuk 2006).

While several studies in Nepal have examined otter habitat preferences and threats (Acharya et al. 2023; Awasthi and Yoxon 2018; Gwachha et al. 2023; Kathariya et al. 2023; Thapa et al. 2020), the majority of work has been confined to protected areas in the lowland Terai. A significant gap still exists on otter research in the western region of Nepal, particularly in the remote and rugged landscapes. The Nepal Otter Action Plan 2020 acknowledges these shortcomings and recommends extensive field surveys for the baseline data on otter population (Thapa 2020). Although otters have been informally reported from the Western Bend of the Karnali River, the area has not yet been the focus of systematic scientific study. It is presumed to support Eurasian otters, with smooth‐coated otters potentially occurring in downstream reaches. Because spraints and pugmarks cannot be reliably assigned to species in the field without molecular confirmation (Khan et al. 2014; Mason and Macdonald 1987; Sharma et al. 2022), all field signs recorded in this study are reported at the genus level as “otters”. This river stretch may therefore constitute important habitat, but the impacts of key threats, including illegal fishing, pollution, and riparian deforestation, remain poorly understood.

As keystone species and top predators, otters play an indispensable role in aquatic ecosystems; their loss could trigger cascading ecological consequences (Fortin et al. 2005; Kruuk 2006). They also serve as flagship species for freshwater conservation, and their presence often indicates good water quality due to their sensitivity to pollution (Kruuk 2006; Mason and Macdonald 1986). Understanding their habitat requirements and the pressures they face is essential for developing effective conservation strategies (Noon et al. 2012). Limited information on otter habitat uses and threats in the Western Bend of the Karnali River makes research in this area critical for conservation. This study examined otter habitat use and conservation threats in this river section, aiming to identify habitat variables associated with otter presence and to assess major threats, treating records of the two potentially sympatric species (Eurasian otter and smooth‐coated otter) collectively at the genus level. The findings of the study provide essential baseline data to inform long‐term monitoring and conservation of otter populations in the region.

## 2 Materials and Methods

### 2.1 Study Area

**FIGURE 1:**
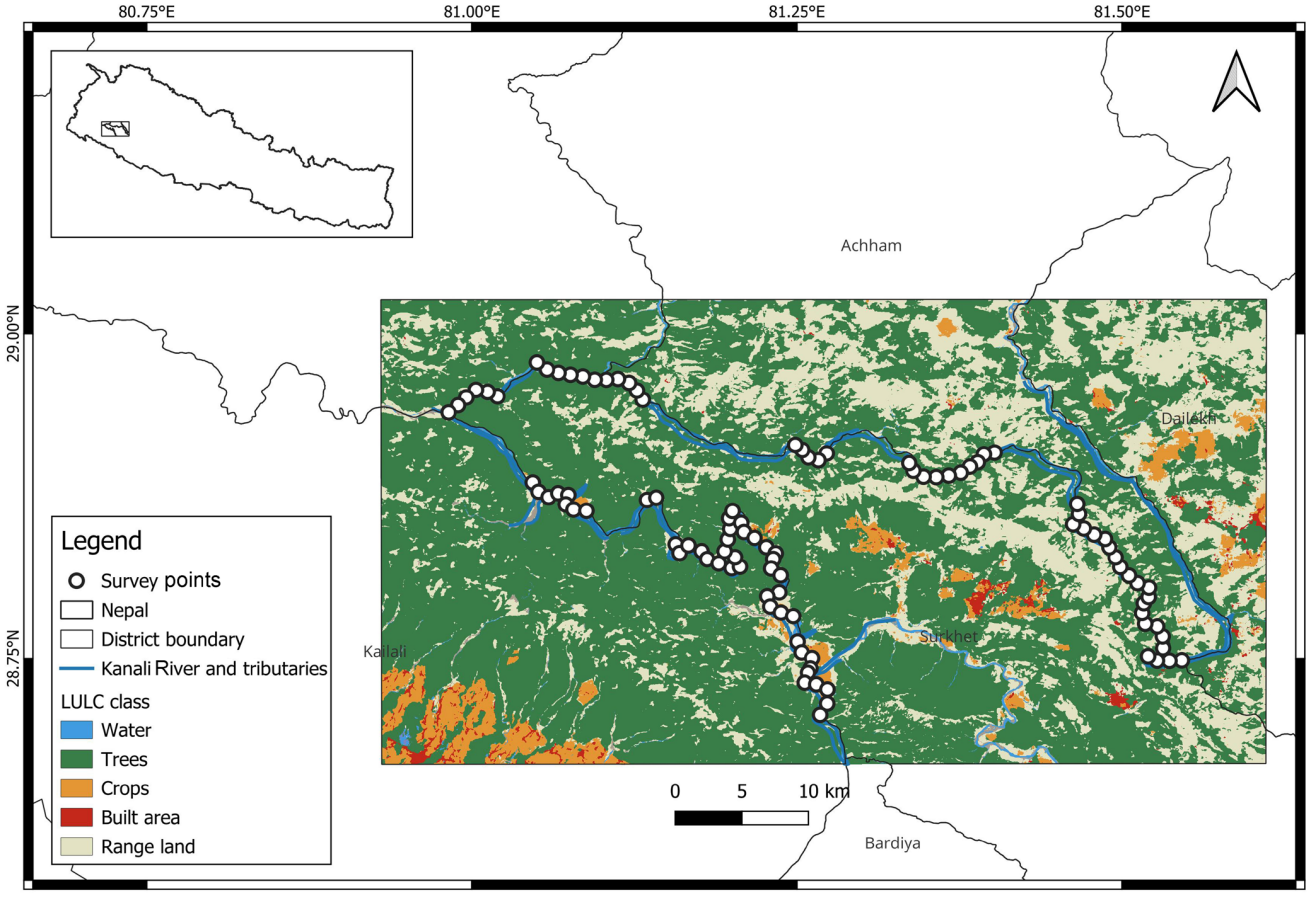
Study area showing the Western Bend of Karnali River with land use classes and survey transects.

This study was conducted along a 150 km section of the Karnali River in western Nepal, from the tri‐border area of Surkhet, Dailekh, and Achham downstream to Kuine, near the border of Bardiya National Park (28°58′40″ to 28°42′25″N and 80°58′55″ to 81°32′48″E) (Figure 1). This stretch of the Karnali River features the distinct “Western Bend,” a sudden directional shift from south to north that forms an approximately 200‐km river bend. This geomorphology helps shape a diverse environment of interconnected riverine systems, including major tributaries like the Seti and Bheri Rivers, wetlands, and bordering forests (McMaster 2019). The basin encompasses diverse landscapes, from the Mahabharat Range to the Dun Valleys, under a subtropical to temperate climate.

Seasonal variations, particularly the monsoon and post‐monsoon periods, significantly impact river hydrology and habitat availability (Dahal et al. 2020; Rolls et al. 2012). The region's vegetation is characterized by riverine forests and grasslands, dominated by species such as Sal ( *Shorea robusta* ) and Khair ( *Acacia catechu* ), which provide essential otter habitat. The basin supports rich biodiversity, including numerous fish species like *Barilius* spp. and *Schizothorax* spp., which are primary prey for otters (Acharya and Paudel 2020; Khatri et al. 2020; Manandhar et al. 2023).

### 2.2 Field Survey

**FIGURE 2:**
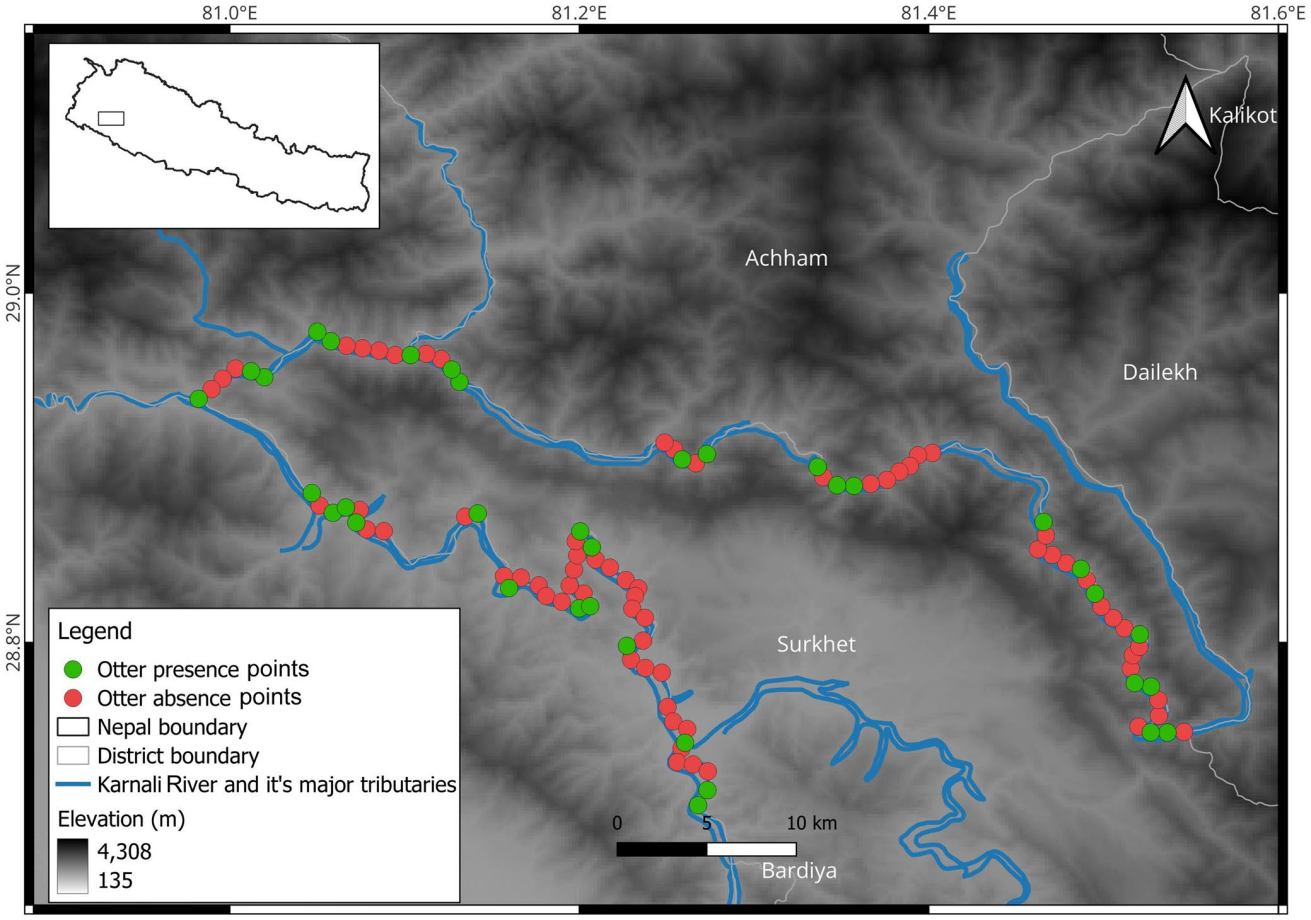
Study area showing the presence and absence of otters along survey transects.

Field surveys were conducted from 28 October to 21 December 2024. This post‐monsoon period was selected as receding water levels expose mud and sandbanks, facilitating the recording of otter signs. Field surveys for otters typically rely on signs such as tracks, scat, latrines, prey remains, and dens (Mason and Macdonald 1987; Wilson and Delahay 2001). In this study, scat distribution served as a proxy for otter distribution (Sittenthaler et al. 2020). The study area was subdivided into 1 km sections along accessible riverbanks on either side of the river, with the surveyed bank at each section determined by terrain accessibility. Each transect included one plot positioned at its starting point, covering 100 m of riverbank and extending 10 m inland from the water's edge. Surveys recorded both indirect evidence of otters (scat, pugmarks, food remains) and direct observations within each plot. A site was considered an otter presence location if either direct observation or indirect signs were recorded. Given the infrequency of otter scats, each occurrence was noted separately, provided that successive scats were spaced more than five meters apart (Melquist and Hornocker 1983; Newman and Griffin 1994). Scats were identified based on prey remains (fish, frogs, crabs, rodents) and a fishy odor (Mason and Macdonald 1987). Field photographs of scats were later verified by research specialists. Some sections of the river could not be surveyed (Figures 1 and 2) due to inaccessible terrain, including steep cliffs and sheer rock faces characteristic of the Western Bend gorge sections. These areas were excluded from the analysis, and gaps in survey coverage should be considered when interpreting the spatial distribution of otter detections.

Water pH was measured using a digital pH meter (pHep, HANNA Instruments, accuracy ±0.1). Water current velocity was estimated by timing a floating ball over a fixed 10 m distance (Gordon et al. 2004). River width was measured with a laser range meter (TM‐1000, 7 × 25 Laser Range Finder). Bank substrate was visually assessed at each transect and classified into four size‐based categories: sand and mud (< 1 cm), small stones (1–10 cm), large stones (11 cm–0.5 m), and rock (> 0.5 m), following predetermined classes (Platts et al. 1983). Mean vegetation cover was estimated at three points (start, middle, end) along each transect and categorized as bare (0%–5%), low (6%–25%), moderate (26%–50%), high (51%–75%), or dense (> 75%), based on Platts et al. (1983).

Within a 100 m radius of each transect, the abundance of dogs, cattle (or cattle tracks), and the presence of trash were assessed. Disturbance levels were graded by observed intensity. Dog abundance was classified as none (0), light (1–3 dogs), moderate (4–6 dogs), or severe (> 6 dogs). Cattle presence/tracks were categorized as none (0), light (1–10), moderate (11–20), or severe (> 20). Trash coverage was evaluated as none (0), light (≤ 5%), moderate (10%), or severe (> 10%) of the 100 m area (Falcone et al. 2010).

Initial field surveys used a handheld range meter to count houses within a 500 m radius to estimate human settlement density (Falcone et al. 2010); these counts were later verified using satellite imagery from Google Earth Pro (Google LLC 2024).

A Human Disturbance Index (HDI) was calculated based on four key threats: (1) habitat loss/fragmentation (HL) from excavation and infrastructure; (2) evidence of otter poaching (PO); (3) fishing evidence (F); and (4) human presence in transects (HP). Each variable was assigned equal weight (0.25) to avoid over‐ or underestimating individual impacts (Limbu et al. 2025; Subba et al. 2024; Pokharel et al. 2022). The HDI for each survey point was computed as: HDI = (HL × 0.25) + (PO × 0.25) + (F × 0.25) + (HP × 0.25). This index reflects the cumulative degree of human disturbance affecting the species' ecology (Falcone et al. 2010).

### 2.3 Data Analysis

Continuous variables were standardized (z‐scores), and categorical variables were converted into factors using ecologically relevant reference categories. Variance Inflation Factors (VIF) were used to check for multicollinearity among predictors. Variables with VIF > 5 were iteratively removed until all remaining predictors exhibited acceptable collinearity levels (Fox and Weisberg 2018).

Given the binary response variable (presence/absence), logistic regression was used to model otter occurrence as a function of habitat variables (Pearce and Ferrier 2000). To reduce overfitting, parameter bias, and model‐selection uncertainty, an information‐theoretic approach with model averaging was adopted instead of a traditional full‐model approach (Burnham 2002). The *dredge* function (*MuMIn* package) selected candidate models, and model‐averaged parameter estimates were derived from models with ΔAICc < 2 (Barton 2023; Aho et al. 2014). Conditional averages and variable importance (sum of Akaike weights) are reported. For ecological interpretation, 95% confidence intervals and odds ratios were calculated (Cumming 2012; Ishwaran and Lu 2019). All analyses were conducted in R 4.3.1 (R Core Team 2023).

## 3 Results

### 3.1 Distribution of Otters in Karnali River

The study area was confirmed to support otters through direct observations and indirect signs (scats, pugmarks, food remains). Otter presence was recorded in 35 of the 102 transects surveyed along the Western Bend of the Karnali River. Direct sightings occurred in 12 transects, while indirect signs (scats and pugmarks) were documented in 27 transects (Figure 2).

Key environmental variables (mean ± SD) across the survey area were: river width (115.2 ± 60.83 m), water current velocity (0.25 ± 0.06 m/s), small stone bank substrate cover (26.25% ± 10.29%), and number of houses within 500 m of the river (5.24 ± 6.97). Among otter presence locations, vegetation cover was predominantly dense (17 sites, 48.58%), human settlement density was high (7 sites, 20.00%) or moderate (9 sites, 25.71%), and distance to settlement was categorized as none in 5 sites (14.29%).

Model averaging of logistic regression models using an information‐theoretic approach revealed six predictors (all continuous predictors were standardized prior to analysis) with consistent effects across all top‐ranked models (ΔAICc < 2) and three predictors with more variable support (Tables 1 and 2). Four models comprised the 95% confidence set, with the top model receiving 38% of the Akaike weight (Table 1).

**TABLE 1:** Top‐ranked models (ΔAICc < 2) predicting species occurrence. Variable codes: 2 = small stone substrate, 4 = dog abundance, 5 = HDI, 6 = house density, 7 = river width, 8 = vegetation cover, 9 = water current velocity.

Rank	*K*	AICc	**Δ**AICc	Weight	Variables
1	10	84.72	0.00	0.38	2,4,6,7,8,9
2	11	85.23	0.51	0.29	2,4,5,6,7,8,9
3	13	86.09	1.38	0.19	2,3,4,5,6,7,8,9
4	11	86.60	1.88	0.15	1,2,4,6,7,8,9

*Note:* ΔAICc = difference from top model; *K* = number of parameters; Weight = Akaike weight.

**TABLE 2:** Model‐averaged parameter estimates (conditional averages) for predictors of species occurrence. All continuous variables are standardized. Reference categories: Dog abundance = High, Vegetation cover = Dense (> 75%), Cattle abundance = High.

Predictor	Estimate	Std error	95% CI	*z*	*p*	Importance	Odds ratio
**Strong and consistent predictors**							
House density (within 500 m)	−6.857	1.905	[−10.64, −3.08]	3.55	< 0.001	1	0.001 [0.000, 0.046]
Vegetation cover: Moderate vs. Dense	5.064	1.661	[1.77, 8.36]	3.01	0.003	1	158.1 [5.88, 4251.8]
Small stone substrate	1.347	0.43	[0.49, 2.20]	3.09	0.002	1	3.85 [1.64, 9.03]
Dog abundance: None vs. Moderate	3.042	1.072	[0.91, 5.17]	2.8	0.005	1	20.94 [2.50, 175.8]
River width	−2.594	0.98	[−4.54, −0.65]	2.61	0.009	1	0.075 [0.011, 0.523]
Water current velocity	−1.228	0.592	[−2.40, −0.05]	2.05	0.041	1	0.293 [0.090, 0.949]
**Moderate predictors**							
Human disturbance index (HDI)	−2.785	1.865	[−6.48, 0.91]	1.48	0.14	0.67	0.062 [0.002, 2.491]
Cattle abundance: Moderate vs. High	−4.511	2.356	[−9.19, 0.17]	1.89	0.059	0.44	0.011 [0.000, 1.186]
**Weak/unclear predictors**							
Cattle abundance: None vs. High	−0.941	1.074	[−3.08, 1.19]	0.86	0.388	0.44	0.390 [0.046, 3.302]
Vegetation cover: Low vs. Dense	2.457	1.578	[−0.68, 5.59]	1.54	0.124	1	11.67 [0.509, 267.5]
Vegetation cover: High vs. Dense	1.23	0.966	[−0.69, 3.15]	1.26	0.209	1	3.422 [0.503, 23.30]
Dog abundance: Moderate vs. Low	1.41	2.452	[−3.44, 6.26]	0.57	0.569	1	4.095 [0.032, 524.8]
Large stone substrate	−0.248	0.318	[−0.88, 0.38]	0.77	0.441	0.19	0.780 [0.415, 1.466]
Intercept	−7.046	1.898	[−10.82, −3.28]	3.66	< 0.001		0.001 [0.000, 0.038]

### 3.2 Key Determinant Variables of Otter Occurrence

**FIGURE 3:**
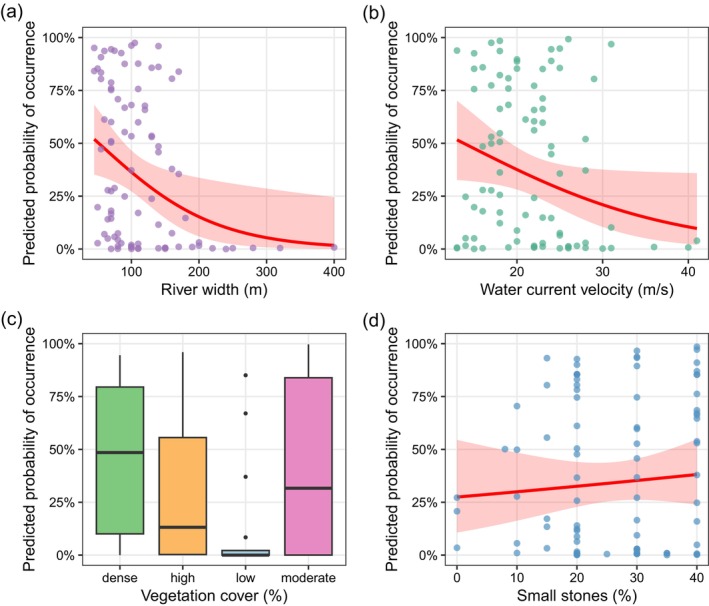
Predicted probability of otter occurrence (%) in relation to (a) river width (m), (b) water current velocity (m/s), (c) vegetation cover (%), and (d) small stones (%). Data indicate habitat suitability for the otters based on environmental variables.

**FIGURE 4:**
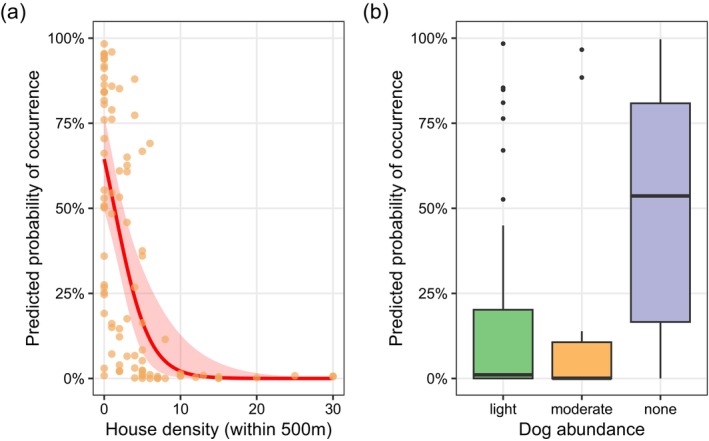
Predicted probability of otter occurrence (%) in relation to (a) house density (within 500 m) and (b) dog abundance. Data indicate habitat suitability for the otters based on threats variables.

Model‐averaged parameter estimates (conditional averages) revealed strong, consistent effects for six variables, all with importance values of 1.00 (included in all top models) and statistical significance at α = 0.05 (Table 2). House density within 500 m exhibited the strongest negative effect (β = −6.857, *p* < 0.001), with each standard deviation increase associated with a 99.9% reduction in occurrence odds (OR = 0.001, 95% CI: 0.000–0.046). River width (β = −2.594, *p* = 0.009; OR = 0.075) and water current velocity (β = −1.228, *p* = 0.041; OR = 0.293) also showed significant negative effects, indicating preference for narrower river sections with slower currents (Figure 3). Moderate vegetation cover (26%–50%) showed the strongest positive effect (β = 5.064, *p* = 0.003), with 158 times higher occurrence odds compared to dense vegetation (> 75%) (OR = 158.1, 95% CI: 5.88–4251.8). Small stone substrate (β = 1.347, *p* = 0.002; OR = 3.85) and absence of dogs (β = 3.042, *p* = 0.005) also significantly increased occurrence probability. Compared to areas with high dog abundance, sites with no dogs had 21 times higher occurrence odds (OR = 20.94, 95% CI: 2.50–175.8, *p* = 0.005) (Figure 4).

The Human Disturbance Index (HDI) showed a negative trend (β = −2.785, *p* = 0.140) consistent with disturbance avoidance, though not statistically significant at α = 0.05. Its inclusion in 67% of top models (importance = 0.67) suggests potential ecological relevance. Moderate cattle abundance exhibited a strong negative effect approaching significance (β = −4.511, *p* = 0.059), with the confidence interval [−9.19, 0.17] including zero, but the point estimate suggesting possible avoidance.

Other vegetation categories (high and low vs. dense) showed positive trends but were not statistically significant (*p* = 0.209 and *p* = 0.124, respectively), with confidence intervals overlapping zero. Large stone substrate (β = −0.248, *p* = 0.441) and absence of cattle (β = −0.941, *p* = 0.388) showed no clear effects on occurrence.

## 4 Discussion

This study confirms the presence of otters in the Western Bend of the Karnali River in western Nepal, an area situated outside the protected area network of the country. The six consistent predictors of otter occurrence identified through our model‐averaging strategy underscore the species' sensitivity to both natural habitat characteristics and anthropogenic disturbances. The strongest effects were from house density (odds ratio = 0.001), indicating extreme avoidance, and moderate vegetation cover (OR = 158.1), indicating strong preference. The nearly complete avoidance of areas near human settlements (OR = 0.001) aligns with numerous studies reporting otter sensitivity to human intrusion. Similar trends have been described in Asia, where otters thrive in sheltered or less disturbed environments but vanish in areas with high human populations (Anoop and Hussain 2004; Kruuk 2006). Tolrà et al. (2024) found that otters avoided high‐accessibility sites, especially near breeding areas, in a heavily anthropized river basin in Spain. Similarly, Nixon et al. (2024) observed that North American river otters avoided locations with dense road networks, likely due to increased disturbance or mortality risk. Our findings, consistent with Dahal et al. (2020) and Raha and Hussain (2016), suggest that human activities such as fishing, sand mining, and settlement expansion degrade habitat quality, reduce prey availability, and consequently restrict otter distribution.

Otters showed a strong preference for moderate vegetation cover (26%–50%), with the odds of occurrence being 158 times higher than in dense vegetation cover (> 75%). This may indicate optimal foraging conditions, where intermediate cover provides both prey habitat and accessibility for hunting. Interestingly, the non‐significant effects of high and low vegetation categories in our study contrast with reports that dense riparian vegetation provides essential shelter and breeding sites (Medina‐Vogel et al. 2003). However, local variation in habitat use is common; Shrestha et al. (2021) found that otter scat abundance was weakly correlated with bank vegetation along the Sanibheri River in Nepal, possibly due to the prevalence of nearly bare riverbanks. Similarly, Romanowski et al. (2013) noted that recolonizing Eurasian otters in Central Poland occupied areas with less tree cover and more managed riverbanks, indicating a degree of habitat tolerance during population recovery.

The preference for small stone substrate (OR = 3.85) is consistent with previous studies on substrate selection by smooth‐coated otters ( *Lutrogale perspicillata* ) and Eurasian otters ( *Lutra lutra* ) for denning and feeding (Nawab and Hussain 2012). However, large stone substrate did not have a significant effect, suggesting that larger habitats may offer fewer prey resources or less suitable burrowing opportunities (Anoop and Hussain 2005). This aligns with Giri et al. (2025), who also found a negative relationship between large stones in the riverbed of Nepal's Myagdi District and otter occurrence.

The negative effects of river width (OR = 0.075) and water current velocity (OR = 0.293) suggest a preference for narrow, slow‐moving streams. Such environments are likely to increase foraging efficiency and reduce energy expenditure. This pattern is consistent with habitat preferences reported for smooth‐coated otters in Asian river systems (Jayasurya et al. 2023; Shenoy et al. 2006), across multiple otter species in Eastern Bhutan (Norbu et al. 2024), and for Eurasian otters in Northern Spain, where otters preferred river reaches with deeper water, abundant aquatic vegetation, and low flow velocities (Tolrà et al. 2024). Discrepancies with studies reporting no width preference may be explained by geographic differences in prey availability or hydrological conditions.

The negative association with dogs (OR = 20.94) identifies domestic and feral canines as key conservation threats. Beyond statistical avoidance, dogs directly cause otter mortality through attacks (Fusillo et al. 2022; Poledník et al. 2011) and act as disease reservoirs (Barros et al. 2022). This combination of predation risk, competition, and pathogen transmission likely explains the extreme avoidance observed in our study and mirrors findings from other regions where otter presence correlates negatively with dog abundance (Prakash et al. 2014; Weston and Stankowich 2014).

Interestingly, while dogs had a strong negative effect, there was no significant difference in the composite Human Disturbance Index (HDI) between otter presence and absence sites. This apparent tolerance of generalized human activity agrees with findings from Nepal (Awasthi et al. 2024) and Europe (Madsen and Prang 2001), indicating that otters can exhibit behavioral adaptability in human‐altered landscapes. However, this resilience appears context‐dependent, as specific disturbances like proximity to houses (OR = 0.001) continue to exert drastic negative impacts. This disparity suggests that otters may tolerate certain anthropogenic pressures while remaining highly sensitive to others, particularly direct aggression from domestic animals.

Management priorities should therefore focus on: (1) establishing dog exclusion zones in key otter habitats, especially during breeding seasons; (2) promoting leash laws and responsible pet ownership in adjacent communities; and (3) monitoring disease transmission between domestic and wildlife populations.

Our cross‐sectional design and single‐season sampling may have missed seasonal variations in otter occurrence. Limited sample sizes for some vegetation categories reduced statistical power to detect threshold effects. Furthermore, the Karnali River Basin is a known important habitat for three species of otter in Nepal (Acharya and Rajbhandari 2011; Jha et al. 2020; Kathariya et al. 2023) and this study was conducted only on a 150‐km section of the river. The pattern of isolated detections interspersed with undetected stretches in the Karnali River basin likely reflects transient habitat use, survey sampling gaps, topographic inaccessibility, and natural habitat patchiness, rather than the absence of otters in intervening areas. Because spraints and pugmarks could not be reliably assigned to species in the field, our records are reported at the genus level; species‐level inference (Eurasian otter vs. smooth‐coated otter) was therefore not possible and would require molecular identification of spraints or camera‐trap confirmation in future work. Future research should investigate the mechanisms behind extreme vegetation preferences, seasonal changes in disturbance response, the experimental impacts of dog disturbance, and landscape‐level connectivity needs.

## 5 Conclusion

This study confirms the occurrence of otters in the Western Bend of Karnali River. Otters in the study section of the Karnali River exhibit strong, multi‐factorial habitat selection shaped by both natural physical templates and anthropogenic pressures. Conservation success will require integrated strategies that address both habitat quality and disturbance reduction, with particular attention to domestic‐animal management. The consistency of these patterns across multiple statistical models provides robust evidence for their ecological importance and underscores the need for proactive conservation measures even outside protected areas.

## Author Contributions

**Madan Acharya:** data curation (equal), formal analysis (equal), investigation (equal), methodology (equal), software (equal), validation (equal), writing – original draft (equal). **Asmit Subba:** data curation (lead), formal analysis (lead), software (equal), writing – original draft (equal). **Naresh Pandey:** investigation (equal), methodology (equal), writing – review and editing (equal). **Laxman Khanal:** conceptualization (lead), resources (equal), supervision (lead), validation (equal), writing – review and editing (lead).

## Funding

This study was supported by the Thesis Research Grant of the Industry, Tourism, Forest and Environment Ministry of Karnali Provincial Government, Nepal.

## Conflicts of Interest

The authors declare no conflicts of interest.

## Data Availability

The data used in this study have been submitted in Science Data Bank (SciDB) and are accessible from the following link: https://doi.org/10.57760/sciencedb.37057.
